# On the processing of optimal performances: Studying arousal evoked by being correct and fast

**DOI:** 10.1002/brb3.2162

**Published:** 2021-05-07

**Authors:** Christian Valt, Birgit Stürmer

**Affiliations:** ^1^ International Psychoanalytic University Berlin Germany

**Keywords:** arousal, correct‐related negativity (CRN), early posterior negativity (EPN), performance monitoring, skin conductance response (SCR)

## Abstract

**Introduction:**

Responses are optimal when they are accurate and fast. The present experiment investigated whether optimal responses evoke physiological arousal and whether performance affects the processing and evaluation of subsequent emotional material.

**Methods:**

Participants performed a response‐choice task, where feedback was a colored square reflecting performance quality or a face whose expression (happy or angry) did not indicate any aspect of performance. In the occurrence of an emotional stimulus, participants had to express a judgment about the emotional strength. The experiment focused on differences in the electrodermal and brain electrophysiological activities evoked by optimal (correct‐fast) and suboptimal (correct‐slow) responses, along with modulations on the processing and interpretation of facial emotions.

**Results:**

The results showed that, compared to correct responses, incorrect responses elicited an augmented phasic skin conductance response (SCR) and enhanced response‐locked event‐related potentials. Importantly, among correct responses, the SCR and the correct‐related negativity (CRN) were larger for correct‐fast than correct‐slow responses. Performance also affected the processing of faces, irrespective of the emotion, but it did not change the subjective interpretation. The EPN evoked by angry and happy faces was less negative after optimal than suboptimal responses.

**Conclusion:**

These results indicate that the monitoring system is sensitive to detect correct‐fast responses, resulting in a state of physiological arousal that might guide the reinforcement of optimal performances.

## Graphical abstract

Optimal performance, like being correct and fast, leads to enhanced response‐related brain activity, increased physiological arousal, and modulations in processing subsequently presented emotional faces. Since these properties are similar to those of error processing, the monitoring system is sensitive to both better‐than‐expected and worse‐than‐expected performance. This sensitivity might lead to learning from the negative reinforcement of errors and the positive reinforcement of optimal responses.

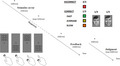

## 1 INTRODUCTION

In many situations, being correct is not sufficient. The speed of correct responses is often an essential requirement for optimal performances and learning. Nevertheless, this aspect has been mostly neglected in the study of the electrophysiological underpinnings of performance monitoring. The present experiment focused on the processing of fast and slow correct responses and investigated whether the detection of optimal responses evokes arousal and influences the processing of subsequent emotional material, at both the electrophysiological and behavioral levels.

A negative event‐related brain potential (ERP) evoked approximately 50 ms after the response over medial fronto‐central recording positions is the first electrophysiological index of performance monitoring based on internal signals. This response‐related negativity reflects the comparison between self‐generated signals associated with the performed action and an action plan (Holroyd & Coles, 2002). Errors induce an increase in the amplitude of this negative potential, called error‐related negativity (Ne/ERN, Falkenstein et al., 1991; Gehring et al., 1993), but a much smaller negative potential is evoked by correct responses as well, the correct‐related negativity (CRN, Vidal et al., 2000). The properties of the Ne/ERN have been extensively investigated since an abnormal amplitude of this ERP has been discussed as an endophenotype of many psychological disorders characterized by internalization (Olvet & Hajcak, 2008; Riesel et al., 2015). The Ne/ERN is associated with increments of defensive reactions and automatic arousal as indexed by the startle potentiation (Hajcak & Foti, 2008; Riesel et al., 2013), the phasic skin conductance response (SCR; Hajcak et al., 2003; O'Keeffe et al., 2004; Paul et al., 2017), and heart rate (Hajcak et al., 2003; Spruit et al., 2018). Moreover, the Ne/ERN primes the processing of negative material. Valt et al. (2017) showed that early visual potentials (P1 and N170) evoked by faces were larger and peaked earlier when an angry face followed an error compared to when it followed a correct response. Moreover, Aarts et al. (2012) showed that errors led to both faster and more accurate categorization of negative words, along with a significant modulation of the early posterior negativity (EPN), an ERP component related to emotion processing (Aarts et al., 2013). The EPN describes a negative potential evoked by emotional stimuli, when compared to neutral stimuli, over parieto‐occipital electrodes at around 250–300 ms (Schupp et al., 2004). This potential is thought to reflect an enhanced perceptual encoding of emotional material induced by the automatic allocation of attention to salient stimuli (Junghofer et al., 2001). In summary, studies of error processing have shown that the amplitude of the Ne/ERN is linked to peripheral physiological responses, probably evoked by the aversive connotations of errors, and that incorrect responses can prime the processing of subsequent negative emotional material.

The Ne/ERN has been the primary focus of studies of performance monitoring. Within this framework, modulations of the CRN have been treated as ERN‐like activities evoked by responses with a negative connotation (Coles et al., 2001). Accordingly, when slow responses are considered erroneous, the CRN is larger for very slow responses than for slow or fast responses (Heldmann et al., 2008; Stahl, 2010). Similarly, in speeded Go/NoGo tasks, where negative feedback followed slow hits, the CRN was larger for slow hits than fast hits (Walentowska et al., 2016). These results suggest that an amplification of the CRN might occur when suboptimal responses result in a negative performance. On a similar line of argumentation, the homogeneity of the source localization and independent component analysis outcomes suggest that the CRN and the Ne/ERN might reflect the same brain activity (Roger et al., 2010). However, a principal component analysis study reported one common factor for the CRN and the Ne/ERN and one factor specific to the Ne/ERN (Endrass et al., 2012). Hence, whether CRN modulations related to response speed monitoring reflect a process characteristic of correct responses or just an interpretation of correct responses as errors is still unclear.

A recent investigation of the CRN functional meaning has called into question the idea that the response‐related negativity (CRN and Ne/ERN) reflects an adverse reaction to an inappropriate performance exclusively. Valt and Stürmer (2017) showed that, regarding the processing of performance speed, the CRN is more negative for fast than slow responses. Considering that correct‐fast responses are preferable compared to correct‐slow responses, the observation of an enhanced CRN for the most desirable performance conflicts with the idea that the CRN is just a smaller Ne/ERN, reflecting the negative connotation of an unfavorable action (Coles et al., 2001). Therefore, the presence of larger negativity for optimal (correct‐fast) than suboptimal (correct‐slow) responses, together with the most substantial negativity evoked by incorrect responses, seems to indicate that the response‐related negativity might reflect a type of processing that is not exclusively related to the aversive feeling of inaccurate actions. This process might be arousal evoked by the positive connotation of correct‐fast responses and by the negative connotation of errors. Arousal describes a valence‐independent activation state of the sympathetic automatic nervous system that leads to increased heart rate, blood pressure, and sweating. In cognitive neuroscience, the electrodermal activity is a standard measurement of arousal (Critchley, 2002). In performance monitoring, Paul et al. (2017) showed that errors concurrently evoke larger response‐related negativity (Ne/ERN) and enhanced SCR (see also Hajcak et al., 2003). Hence, the observation of larger CRN for correct‐fast than correct‐slow responses (Valt & Stürmer, 2017) might also be linked to an enhancement of phasic arousal in trials with optimal performances.

Since arousal describes a state of physiological activation, optimal responses might influence the processing of subsequent stimuli, particularly when they incorporate an emotion. Arousal is a key factor in many models of emotion processing (Moors, 2009). In line with the potential role of arousal for the processing and interpretation of emotions, in Valt and Stürmer (2018), the EPN evoked by smiling faces was less negative in trials where the feedback indicated a correct‐fast response compared to trials where the feedback indicated a correct‐slow or a correct‐average response. This modulation occurred for smiling faces irrespective of the emotional valence expressed by the eyes (happy, neutral, or sad). Moreover, participants judged faces happier when they appeared in trials with correct‐fast responses compared to when they appeared in trials with correct responses made with average or slow speed. Based on these results, optimal performances seem to affect also the processing and interpretation of smiling faces, and arousal might be the mediator of such an influence of performance on emotion processing. Since arousal is an unspecific valence‐independent response (Russell, 1980), the physiological activation potentially evoked by an optimal performance might affect the processing and interpretation of facial expressions irrespective of whether the emotion is positive (happy) or negative (angry).

The described CRN studies conducted in our laboratory (Valt & Stürmer, 2017, 2018) left two open questions. Are optimal responses associated with phasic arousal? Do optimal responses prime only the processing of happy faces? The present experiment aimed to answer these questions by testing the hypothesis that optimal responses evoke physiological arousal, resulting in modulations of the processing of subsequent emotional material, irrespective of its valence. To this end, we recorded the electrodermal and electrophysiological response evoked by incorrect, correct‐fast, or correct‐slow responses and explored the potential influences that performance might have on the processing and evaluation of an unrelated emotional face. Participants performed a response‐choice task with feedback. The external signal was a red square after errors, while correct responses were followed, in 1/3 of the trials, by a colored square, used as informative feedback of response speed (green for fast, olive‐green for average speed, and orange for slow), or, in 2/3 of the trials, by a face with a happy or an angry expression. Importantly, the emotion of the face was unrelated to response speed, and participants had to express a judgment on the strength of the emotion.

The present experiment should clarify whether the detection of an optimal performance based on internal signals evokes physiological arousal. In line with Paul et al. (2017), the SCR evoked by responses should be larger in incorrect than correct trials. According to the hypothesis that optimal responses also elicit phasic arousal, correct‐fast responses should also show an enhanced SCR starting 2 s after the response (see, Hajcak et al., 2003). Since the SCR is a slow physiological response that requires a minimum of 1 s to build‐up, in an experimental design with fast sequences of events, like the present one, the investigation of arousal evoked by response monitoring requires the ruling out of potential influences of earlier or later processing. Therefore, we performed two separate statistical analyses to control for the independence of outcomes related to internal signal processing from other possible sources. In the first analysis, we controlled for feedback in the current trial. Since colored squares were informative feedback of response speed and accuracy, potential SCR differences between correct‐fast and correct‐slow responses might reflect the processing of informative external signals. We controlled for this possibility by analyzing whether informative or uninformative feedback modulated the SCR evoked by correct‐fast and correct‐slow responses. On the one hand, if the detection of an optimal response based on internal signals is sufficient to elicit arousal, we should observe a significant main effect of response speed also in the conditions where the feedback is uninformative. On the other hand, if the additional support of informative feedback is necessary for triggering arousal, the results should reveal a significant interaction of performance with feedback, indicating that the difference between correct‐fast and correct‐slow responses was present only in trials with colored squares. In the second analysis, we controlled for feedback in the previous trial because, in trials where a face followed the response, participants had to make an emotionality judgment that could induce a slow‐down of response times. Hence, potential SCR differences between correct‐fast and correct‐slow responses might reflect residual processing from the previous trial or interference caused by switching from the mouse to the response buttons. To control for this possibility, we took into account the nature of the feedback in the previous trial. On the one hand, if performance monitoring based on internal signals is the principal cause of SCR modulations, we should observe a significant difference between correct‐fast and correct‐slow responses irrespective of the previous feedback. On the other hand, the observation of a significant influence of preceding feedback would indicate that the emotionality judgment in the previous trial determined a change of participant's arousal irrespective of whether the given response was fast or slow.

At the brain electrophysiological level, in agreement with previous studies, compared to suboptimal responses, optimal responses should elicit a more negative CRN (Valt & Stürmer, 2017), and they should modulate the brain response evoked by faces (Valt & Stürmer, 2018). In the response‐locked analysis, we did not expect any significant effect from the previous feedback because of the delay between trials. Nonetheless, we considered feedback in the previous trial both in the analysis of the response‐locked activity and the processing and interpretation of faces. In the processing of faces, we expected to observe a less negative EPN in trials with correct‐fast responses compared to trials with correct‐slow responses. The presentation of both angry and happy faces should inform whether the effect of performance on emotion processing is linked to positive valence, with modulations occurring only in the processing of smiling faces, or it is independent of valence, with modulations affecting both happy and angry faces. The effect of performance on face processing should also determine a correspondent change in the subjective judgments on emotional strength.

To summarize, the present experiment employed a response‐choice task with feedback to investigate whether the detection of optimal responses, here considered as responses that are both accurate and fast, generates physiological arousal and affects the processing and interpretation of unrelated happy or angry faces.

## 2 METHOD

### 2.1 Participants

Twenty‐eight participants (13 men and 15 women) took part in the experiment. The mean age of the participants was 25 years (ranging from 20 to 53). A power analysis conducted with the software G‐Power showed that, based on the effect size of the CRN contrast between correct‐fast and correct‐slow responses in Valt and Stürmer (2017), twenty participants were necessary to achieve a power of 0.95 (*α* = 0.05, two‐tailed). Hence, the present sample size was sufficient for the replication of this key electrophysiological effect, offering a reliable starting point for the employed electrodermal analysis.

All participants had a normal or corrected‐to‐normal vision, and, according to the Edinburgh Handedness Inventory (Oldfield, 1971), 20 participants were right‐handed, seven participants were left‐handed, and one participant was ambidextrous. The Ethics Committee of the International Psychoanalytic University Berlin approved the study and, before the beginning of the preparation for the experiment, participants gave their informed consent. At the conclusion of the experiment, participants received 20 € or course credits for their attendance in the study.

### 2.2 Procedure

**FIGURE 1:**
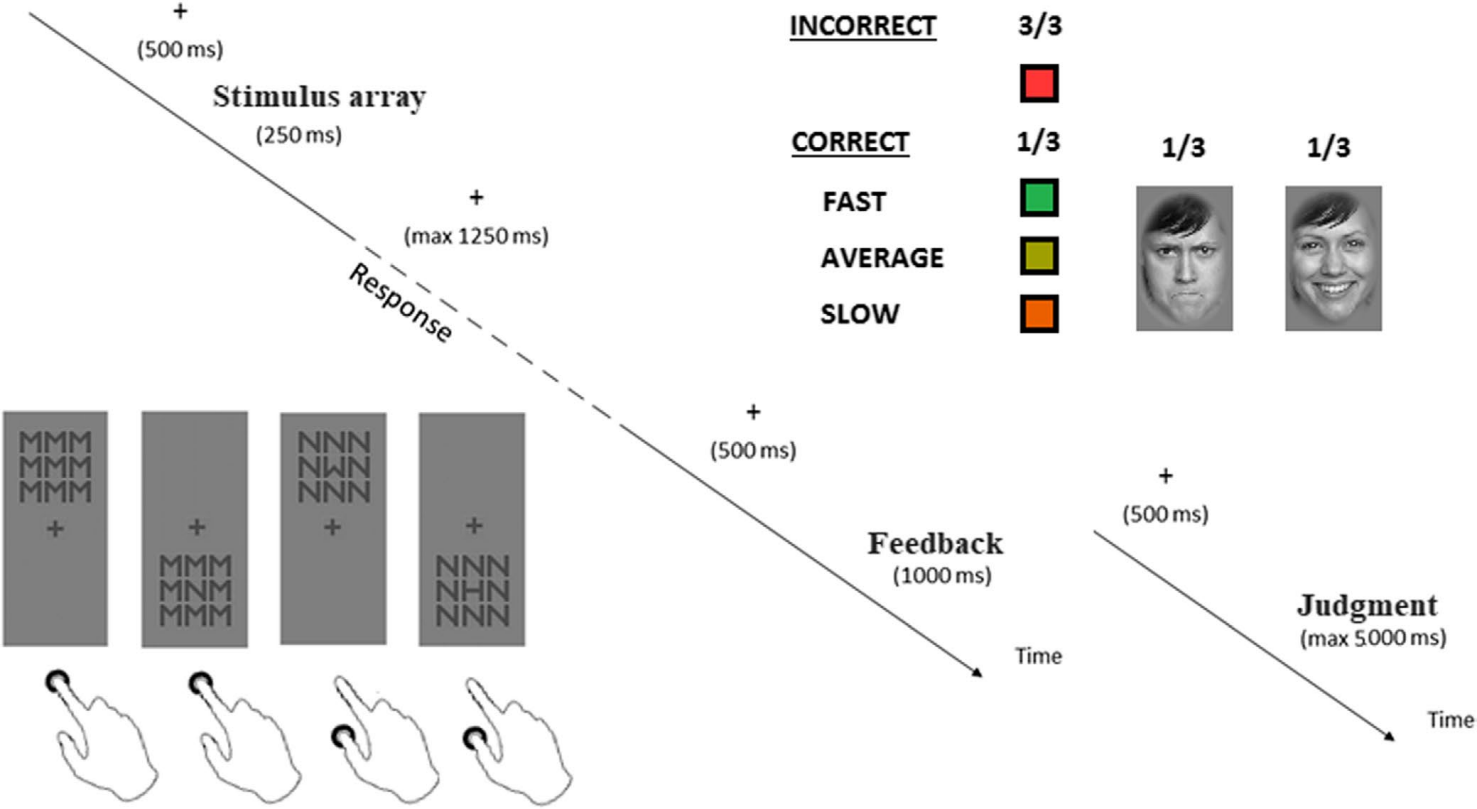
Schematic representation of the response‐choice task, the time course of trials, and the stimuli used as feedback of incorrect and correct responses

Participants performed a response‐choice task on the identity of the central letter in a 3 × 3 array of letters. The letters M, N, W, and H formed the arrays with one letter presented in the center and one letter placed eight times around the center. Letters were assigned to two response buttons arranged vertically on the desk. For example (see Figure 1), participants had to press with the right index finger the upper button when the target letter was M or N, and the lower button, with the right thumb, when the target letter was H or W. The mapping of letters to response buttons changed across participants. The target letter could be identical to or different from the surrounding letters resulting in sixteen different arrays. The congruency or incongruency between the response required by the target letter and the response associated with the flanker letters determined an Eriksen conflict (Eriksen & Eriksen, 1974). Stimulus arrays could appear above or below the fixation cross resulting in two stimulation positions. The congruency or incongruency between the relative position of the stimulus array on screen and the position of the response buttons on desk determined a Simon conflict (Simon, 1969).

The study started with three practice blocks, followed by sixteen experimental blocks. Each block had 32 trials (16 stimulus arrays displayed in two stimulation positions) consisting of one stimulus array followed, in one third of correct trials and in all incorrect trials, by a colored square or, in two thirds of correct trials, by a face (see Figure 1). Squares had a red (RGB: 255, 53, 53), green (RGB: 35, 177, 77), olive‐green (RGB: 159, 159, 1), or orange (RGB: 235, 95, 0) color according to the quality of the response. Red squares were feedback of incorrect responses, whereas, green, olive‐green, and orange squares were feedback of the speed of correct responses, computed based on the eighth fastest and slowest RTs in the previous 24 correct trials. The green, olive‐green, and orange squares appeared in trials with fast (RT ≤ eighth fastest response), average (eighth fastest response > RT ≤ eighth slowest response), or slow responses (RT > eighth slowest response), respectively. Therefore, colored squares were accurate feedback of performance. Faces were black‐and‐white portraits of 160 people with a happy or an angry expression, taken from the stimulus set FACES (Ebner et al., 2010). Contrary to colored squares, the expression of the faces was not feedback of any aspect of performance because happy and angry faces appeared in two‐thirds of correct trials irrespective of response speed. In practice trials, responses were followed only by colored squares, to practice the analysis of response accuracy and speed.

For each face, participants had to report the strength of the emotion. They had to indicate their subjective evaluation by moving with the computer mouse an arrow along a bar presented in the center of the screen. The bar was 768 pixel long, with the left and the right extremities meaning weak and strong emotion, respectively. Instructions invited the participants to use the whole length of the bar and make precise judgments.

Each trial (see Figure 1) started with the presentation of a fixation cross in the middle of the screen for 500 ms. Afterward, the stimulus array appeared above or below the fixation cross for 250 ms. The response period started at the offset of the stimulus array, resulting in the exclusion of responses that were too fast (RTs <250 ms). During the 1,250 ms maximum duration of the response period, the fixation cross was the only object on screen, and the program recorded the participant's reactions. The fixation cross remained on screen for further 500 ms after the response or after the conclusion of the response period, and the colored square or the emotional face was then displayed for 1 s. When the external signal was an emotional face, at face offset, participants had a maximum time of 5 s to make the judgment. The next trial started after 500 ms of blank screen.

The fixation cross and single letters in the array had a dark gray color (RGB: 78, 78, 78) and a size of 0.32° × 0.32° of visual angle. The stimulus array could appear above or below the fixation cross with a center‐to‐center distance of 0.80°, and gaps of 0.05° divided the letters within the array. Colored squares had a size of 1.20° × 1.20°; whereas faces fitted in a rectangular shape with rounded edges and a size of 6.81° × 4.52°. Throughout the study, the background color was light gray (RGB: 128, 128, 128).

Before the beginning of the first experimental blocks, instructions informed the participants that, throughout the experiment, errors and slow responses were punished by the subtraction of 0.05 € and 0.02 €, respectively, from a starting bonus of 15.00 €. This procedure was adopted to incentivize a constant focus on the task. At the end of each run of four experimental blocks, participants were informed about the amount of money left in the bonus, which was then granted at the conclusion of the experiment. Moreover, written feedback presented at the end of each run encouraged the participant to be faster or more accurate if the number of errors in the last four experimental blocks distanced substantially from the ideal error rate of 10% (less than 5 or more than 20 errors in the four blocks).

### 2.3 Data processing

#### 2.3.1 Electrodermal activity

Ag/AgCl electrodes placed on the palmar surface of the left hand measured the electrodermal activity (EDA). Participants had to place the left hand in a comfortable position on the desk, and they had to keep the hand motionless during the recording. The EDA electrodes were connected to an ExG amplifier (BrainProduct GmbH) that generated a constant voltage of 0.5 V and recorded the signal in direct current (DC) mode. The EDA recording used the same parameters of the EEG recording, with a resolution of 152.6 μV and a range of 5000± mV.

EDA data were analyzed with the MATLAB‐based software Ledalab (http://www.ledalab.de/; Benedek & Kaernbach, 2010). The EDA was first down‐sampled to 20 recordings per second (as recommended by Ledalab tutorial: www.ledalab.de/decumentation.htm) and then visually inspected for artifacts. Two participants were not considered in the EDA analysis because of too many artifacts; whereas, for three participants, segments with artifacts were rejected. In conformity to the procedure used by Paul et al. (2017), the signal was first filtered (low‐pass Butterworth filter of 5 Hz) and then smoothed (convolution with an eight‐point Gaussian window). Afterward, the phasic SCR values evoked by errors, correct‐fast, and correct‐slow responses were extracted in the time window 0.5–3.5 s after response (minimum amplitude criterion: 0.05 μS). We divided the 3 s interval into two 1.5 s time windows (early: 0.5–2.0 s; late: 2.0–3.5 s) to obtain a better temporal resolution, since in Hajcak et al. (2003) the effect seemed to start 2 s after the response. Finally, each individual SCR value was range‐corrected (SCR value/mean [SCRmax value−SCRmin value]).

#### 2.3.2 Electrophysiological activity

Sixty‐four Ag/AgCl electrodes mounted in an elastic electrode cap and two Ag/AgCl electrodes applied directly to the skin over the left and right mastoids, M1 and M2, recoded the EEG with the software BrainVision Recorder. According to the International 10/20 System, the locations of the electrodes in the cap corresponded to the positions: Fp1/2, Fpz, AF7/8, AF3/4, F9/10, F7/8, F5/6, F3/4, Fz, FT9/10, FC5/6, FC3/4, FC1/2, FCz, T7/8, C5/6, C3/4, Cz, TP9/10, CP5/6, CP3/4, CP1/2, CPz, P9/10, P7/8, P5/6, P3/4, Pz, PO9/10, PO7/8, PO3/4, POz, O1/2, Oz, Iz. The initial common reference was M1, and the ground was AFz. One Ag/AgCl electrode placed at the outer canthi of the right eye (horizontal) and one Ag/AgCl electrode placed below the right eye (vertical) recorded the electrooculogram (EOG). EEG and EOG signals were digitalized with a frequency of 500 Hz and a band‐pass filter of 0.05–70 Hz. Electrodes’ impedance was kept below 5 kΩ for all the electrodes.

Offline, the EEG signal was processed with BrainVision Analyzer 2.1. EEG data were first filtered with a low‐pass filter of 30 Hz (slope of 48 dB/octave) and then corrected from blinks, eye‐movements, and pulse artifacts with independent component analysis trained on calibration trials performed at the end of the experiment. After this preprocessing, the EEG signal was segmented to create response‐locked epochs for trials with incorrect, correct‐fast, or correct‐slow responses, and face‐locked epochs for trials with correct‐fast or correct‐slow responses. Epochs started 200 ms before response or face onset and lasted for 400 ms, when response‐locked, or 600 ms, when face‐locked. Based on visual inspection, an average of 2.8% of epochs was discarded from further analyses because of artifacts. All epochs without artifacts were aligned to the 200‐ms period preceding response or face onset and rereferenced to the average activity of all electrodes (with the exclusion of the electrodes PO9/10, POz, and Iz for balancing reasons). The response‐related negativity (Ne/ERN and CRN) was computed as the average activity of the response‐locked ERPs at electrode Fz between 0 and 75 ms. The electrode and time window are standard selections in the literature, adopted to conform to the parameters used in Valt and Stürmer (2017). The EPN was computed as average activity evoked by happy and angry faces between 200 and 300 ms at electrodes P10/9, P8/7, PO10/9, and PO8/7. The selection of the electrodes and the time window for the calculation of the EPN was based on the recording of the brain activity evoked by happy, angry, and neutral faces (60 stimuli for each emotion) presented in random sequence at the end of the experiment for passive viewing. During the passive viewing task, faces were presented for 1,000 ms and divided by fixation periods of 500 ms.

#### 2.3.3 Subjective judgment of emotion strength

The subjective judgments of emotional strength were converted from continuous (from −384 pixels to 384 pixels) to discrete values, by dividing the length of the bar into eight equal‐sized categories (0–7). This procedure was employed to account for small differences between continuous judgments.

### 2.4 Data analysis

Among trials with correct responses, we considered only trials with correct‐fast and correct‐slow responses because of the uncertainty present in responses made with average speed (Valt & Stürmer, 2017). This procedure should maximize performance classification based on internal signals.

We first contrasted correct‐fast and correct‐slow responses against incorrect responses, to check for the adequacy of the present design in detecting performance‐related SCR and ERP effects. Afterward, we focused on the contrast between correct‐fast and correct‐slow responses. Here, we also checked for potential influences from feedback in the current trial (SCR analysis) and feedback in the previous trial (SCR, response‐locked ERP, and face‐locked ERP analyses). For these analyses, incorrect trials were not considered because the larger responses evoked by incorrect trials might have masked subtle dynamics of correct‐fast and correct‐slow responses.

We started the analysis of arousal by dividing correct‐fast and correct‐slow responses according to the type of feedback in the current trial. In this analysis, the statistical test considered the factors Performance (correct‐fast and correct‐slow), Current Feedback (colored square, happy face, and angry face), and SCR Time Window (early and late).

For all the other analysis, we divided segments according to feedback in the preceding trial. The decision to take into account the feedback condition in the previous trial imposed the exclusion from the analysis of the response‐related activity of trials that followed a break between blocks. Trials that followed an incorrect response were also not considered in this analysis to keep an equal number of trials in the different conditions and rule out potential confounds from error processing in the previous trial. Despite these strict exclusion criteria, we had sufficient trials for the calculation of reliable ERPs (Olvet & Hajcak, 2009). The average number of correct‐fast trials was 52 after squares, 38 after angry faces, and 39 after happy faces; and the average number of correct‐slow trials was 32 after squares, 42 after angry faces, and 41 after happy faces. In these analyses, the considered factors were Performance (correct‐slow, and correct‐fast) and Preceding Feedback (colored square, happy face, and angry face), along with the factor SCR Time Window (early or late) for physiological arousal, and Emotion (happy and angry) for ERP and behavioral effects related to angry and happy faces.

Statistical analyses were performed with repeated‐measurements ANOVAs. Follow‐up related‐samples two‐tailed *t* tests explored the direction of significant main effects. The significance level of the ANOVAs and post hoc *t* tests was *α* = .05. For exploratory *t* tests, the significance level was adjusted with Bonferroni correction to *α*/3 = .017.

## 3 RESULTS

### 3.1 Behavioral performance

After the exclusion of 1.13% (*SE* = 0.3%) of the trials because the participant did not react to the letter array within the response period or the response time was faster than 250 ms, the mean error rate was 12.10% (*SE* = 1.60%).

Two ANOVAs investigated the factor Preceding Feedback (colored square, happy face, or angry face) on accuracy and reaction times. The nature of feedback presented in the preceding trial did not affect the mean accuracy, *F* < 1, but determined a significant effect on RTs, *F*(2, 54) = 45.10, *p* < .001, *η^2^_p_* = .626. Participants were significantly faster when the N‐1 feedback was a colored square compared to when it was a happy or an angry face, *t*(27) = 7.19, *p* < .001, *d* = 1.42, and *t*(27) = 7.56, *p* < .001, *d* = 1.36, respectively, but RTs did not differ significantly in trials where the N‐1 feedback was a face, *t*(27) = 1.81, *p* = .081. This result reflected a slowing‐down of RTs caused by switching from the mouse, for the judgment of emotional strength, to the response buttons, for the response‐choice task.

RTs of incorrect responses (*M* = 683 ms, *SE* = 21) were significantly slower than RTs of correct‐fast responses (*M* = 591 ms, *SE* = 13), *t*(27) = 9.52, *p* < .001, *d* = 1.80, and significantly faster than RTs of correct‐slow responses (*M* = 831 ms, *SE* = 21), *t*(27) = 13.09, *p* < .001, *d* = 2.47.

### 3.2 Response‐locked SCR

**FIGURE 2:**
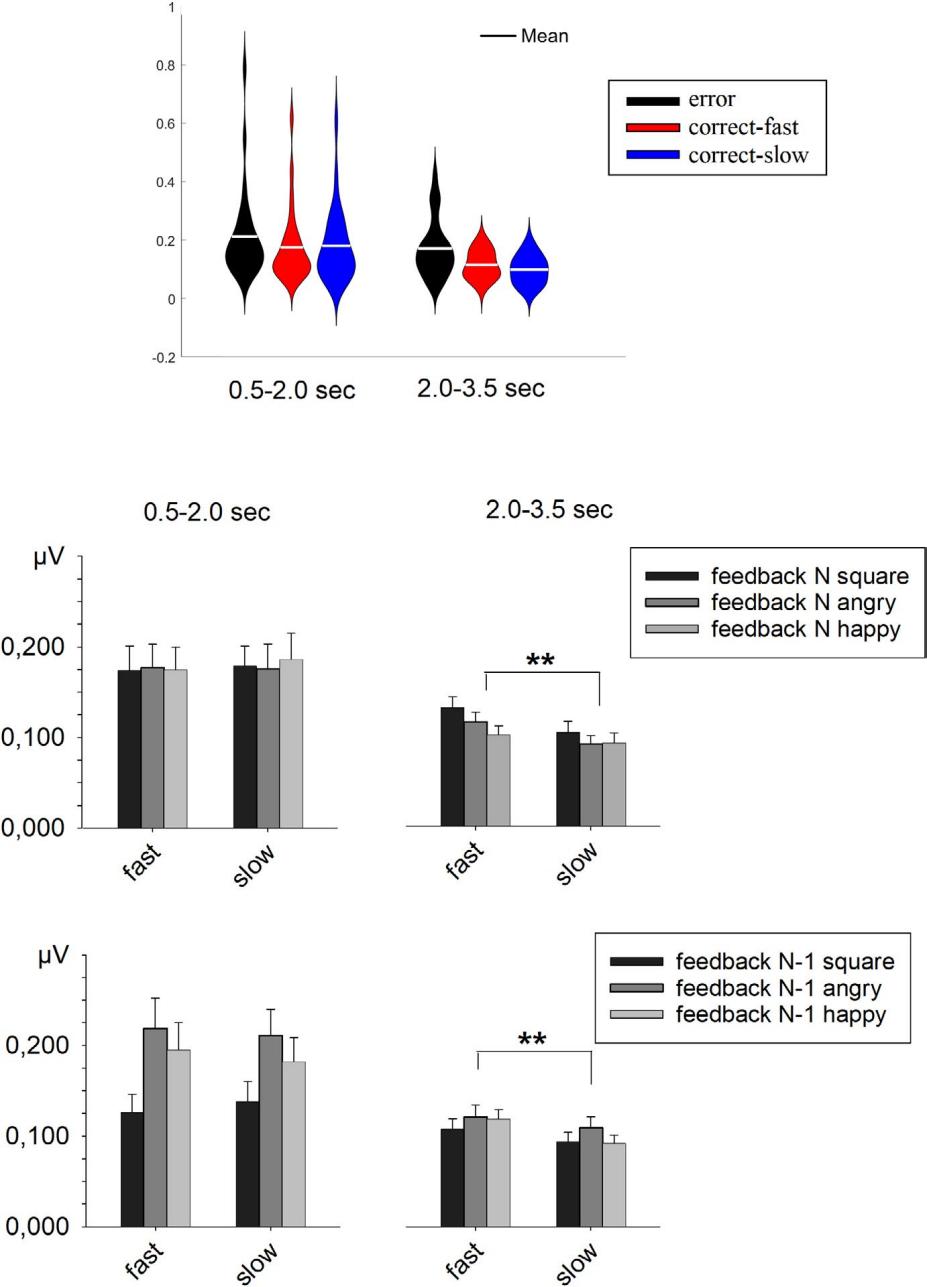
The violin plot depicts the distribution of individual data points in the three performance conditions. The bar plots describe the SCR mean activity in the early and the late time window. The upper bar plots depict the mean SCR activity of trials divided according to feedback in the current trial (feedback N); the lower bar plots depict the mean SCR activity of trials divided according to feedback in the previous trial (feedback N‐1)

Errors evoked a significantly larger SCR response than correct‐fast and correct‐slow responses in both the early time window, *t*(25) = 3.22, *p* = .004, *d* = 0.63, and *t*(25) = 2.74, *p* = .011, *d* = 0.54, and the late time window, *t*(25) = 2.69, *p* = .013, *d* = 0.52, and *t*(25) = 3.52, *p* = .002, *d* = 0.69 (see Figure 2). These results conformed to previous SCR studies (Hajcak et al., 2003; Paul et al., 2017) that reported enhanced arousal for incorrect responses, suggesting that the present experiment was suitable for detecting performance‐related physiological activations.

The ANOVA performed on the SCR evoked by correct‐fast and correct‐slow responses in the two time windows showed a significant interaction between these two factors, *F*(1, 25) = 7.36, *p* = .012, *η* ^2^ *_p_* = .227 (see Figure 2). This interaction revealed that the SCR was larger for correct‐fast than correct‐slow responses in the late time window, *t*(25) = 3.27, *p* = .003, *d* = 0.64, but the difference was not significant in the early time window, *t*(25) = 0.72, *p* = .48. Additional checks were conducted to explore the potential influences of feedback in the current trial and feedback in the preceding trial on the SCR modulations induced by optimal responses.

To control whether informative feedback influenced the SCR, an ANOVA contrasted correct‐fast and correct‐slow responses by taking into account feedback in the current trial (see Figure 2). The results revealed a significant main effect of SCR Time Window, *F*(1, 25) = 7.45, *p* = .011, *η* ^2^ *_p_* = .230, meaning that the SCR was larger in the early than the late time window, but no significant main effects of Current Feedback, *F*(2, 50) = 1.02, *p* = .367, and Performance, *F*(1, 25) = 2.57, *p* = .122. Interestingly, the results revealed a significant interaction between SCR Time Window and Performance, *F*(1, 25) = 8.10, *p* = .009, *η* ^2^ *_p_* = .245. For the other interactions, there was a trend toward a significant interaction between SCR Time Window and Current Feedback, *F*(2, 50) = 2.59, *p* = .085, but no significant interaction between Performance and Current Feedback, *F*(2, 50) = 0.62, *p* = .541, and among the three factors, *F*(2, 50) = 0.19, *p* = .824. The interaction between SCR Time Window and Performance indicated that the SCRs evoked by correct‐fast and correct‐slow responses did not show any significant difference in the early time window, *F*(1, 25) = .55, *p* = .464, but correct‐fast responses elicited a significantly stronger SCR in the late time window, *F*(1, 25) = 12.73, *p* = .001, *η* ^2^ *_p_* = .337. In both time windows, there was no significant interaction between Performance and Current Feedback, *F*s(2, 50) < 1.25, *p*s > .296. The lack of such a significant interaction indicated that the SCR enhancement for correct‐fast responses was similar among the three feedback conditions. The evidence of a significant effect of performance also when the analysis was restricted to the two conditions with uninformative feedback, *F*(1, 25) = 6.24, *p* = .019, *η* ^2^ *_p_* = .200, supplied convincing evidence that informative feedback was not determinant for the observed SCR result. These outcomes indicated that the detection of an optimal response based on internal signals is sufficient for the activation of arousal.

To control for influences from processing in the previous trial, particularly when the participant had to express the emotionality judgment, we then considered feedback in the preceding trial as a splitting factor (see Figure 2). The main effects were all significant: SCR Time Window, *F*(1, 25) = 7.07, *p* = .013, *η* ^2^ *_p_* = .221, Preceding Feedback, *F*(2, 50) = 15.04, *p* < .001, *η* ^2^ *_p_* = .363, and Performance, *F*(1, 25) = 4.87, *p* = .037, *η* ^2^ *_p_* = .163. SCR Time Window interacted significantly with Preceding Feedback, *F*(2, 50) = 7.67, *p* < .001, *η* ^2^ *_p_* = .235, but the interaction with Performance was short of significance, *F*(1, 25) = 4.11, *p* = .054, *η* ^2^ *_p_* = .141. The interaction between Preceding Feedback and Performance was not significant, *F*(2, 50) = 1.06, *p* = .356, and the three‐way interaction was also not significant, *F*(2, 50) = 0.60, *p* = .555. The significant interaction between SCR Time Window and Preceding Feedback indicated that feedback in the previous trial had a significant effect in the early time window, *F*(2, 50) = 14.26, *p* < .001, *η* ^2^ *_p_* = .363, but not in the late time window, *F*(2, 50) = 1.31, *p* = .278. In the early time window, the SCR was smallest after squares and largest after angry faces. The nearly significant interaction between SCR Time Window and Performance indicated that the SCR amplitude difference between correct‐fast and correct‐slow response was not significant in the early time window, *t*(25) = 0.43, *p* = .674, but significant in the late time window, *t*(25) = 3.41, *p* = .002, *d* = 0.67.

### 3.3 Response‐locked ERP

Errors evoked a significantly larger response‐related negativity than correct‐fast and correct‐slow responses, *t*(27) = 2.69, *p* = .012, *d* = 0.51, and *t*(27) = 3.99, *p* < .001, *d* = 0.75, respectively. Importantly, the CRN evoked by correct‐fast responses was significantly larger than the CRN evoked by correct‐slow responses, *t*(27) = 2.71, *p* = .011, *d* = 0.51.

**FIGURE 3:**
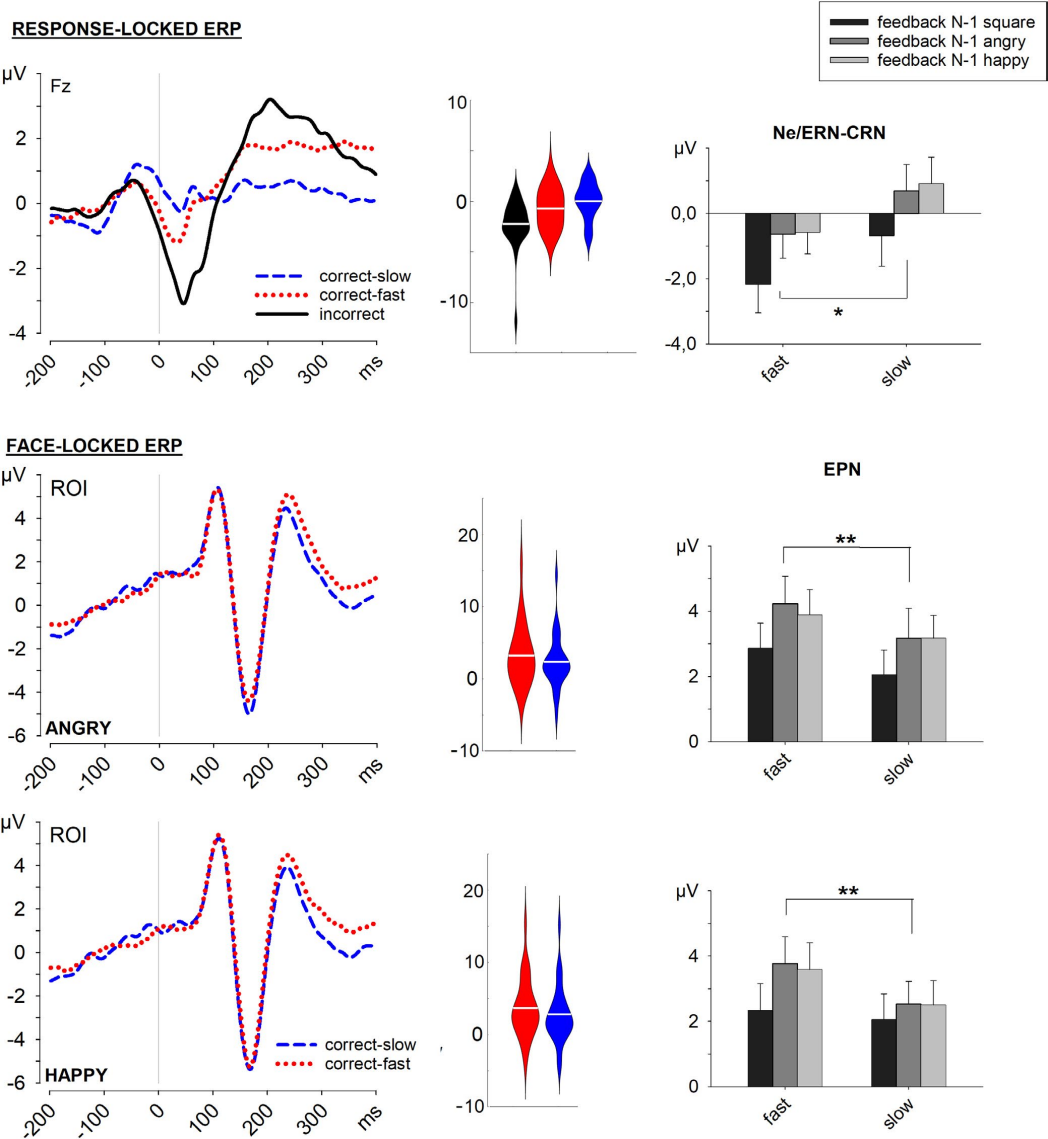
Response‐locked grand average ERPs at Fz and face‐locked grand average ERPs in the ROI (P10/9, P8/7, PO10/9, and PO8/7) for angry and happy faces. The violin plots depict the distributions of individual data points in the different performance conditions. The bar plots describe the mean activity of the response‐related negativity (Ne/ERN‐CRN) in the time window 0–75 ms and the mean EPN in the time window 200–300 ms, separately according to the N‐1 feedback

The ANOVA performed on the average amplitudes of the CRN evoked by correct‐fast and correct‐slow responses (see Figure 3) showed a significant main effect of Performance, *F*(1, 27) = 5.52, *p* = .026, *η* ^2^ *_p_* = .170, and a significant main effect of Preceding Feedback, *F*(2, 54) = 14.09, *p* < .001, *η* ^2^ *_p_* = .343. The main effect of Preceding Feedback revealed more negative response‐related negativities in trials that followed colored squares compared to trials that followed angry, *t*(27) = 3.86, *p* < .001, *d* = 0.73, or happy faces, *t*(27) = 4.34, *p* < .001, *d* = 0.82; but the difference after happy and angry faces was not significant, *t*(27) = 1.19, *p* = .25. Since the interaction between these two factors was not significant, *F* < 1, these results indicated that it was unlikely that the observed effect of performance on the CRN was determined by feedback in the previous trial.

The correlation between ΔSCR (correct‐fast SCR—correct‐slow SCR) and ΔCRN (correct‐fast CRN—correct‐slow CRN) was not significant, *r* = .125, *p* = .540.

### 3.4 Face‐locked ERP

In the analysis of the amplitude of the EPN, all three main effects were significant: Preceding Feedback, *F*(2, 54) = 18.83, *p* < .001, *η* ^2^ *_p_* = .411, Emotion, *F*(1, 27) = 10.33, *p* = .003, *η* ^2^ *_p_* = .277, and Performance, *F*(1, 27) = 12.19, *p* = .002, *η* ^2^ *_p_* = .311. No interaction among these factors was significant, *F*s < 1.76, *p*s > .182. The main effect of Preceding Feedback revealed an overall smaller EPN amplitude when the N‐1 feedback was a colored square compared to a happy or an angry face, *t*(27) = 4.58, *p* < .001, *d* = 0.87, and *t*(27) = 5.17, *p* < .001, *d* = 0.98, respectively, but no difference when the N‐1 feedback was a happy or an angry face, *t*(27) = .86, *p* = .397. The main effect of Emotion indicated a smaller EPN amplitude in the processing of happy faces. Importantly, Performance was also significant as a main effect, reflecting a less negative potential in trials with correct‐fast responses than in trials with correct‐slow responses (see Figure 3). In the absence of any significant interaction, the present results suggest that, compared to correct‐slow responses, correct‐fast responses induced an enhancement of the brain response evoked by faces, irrespective of the emotion.

### 3.5 Subjective judgment

The average rating given to angry faces was *M* = 4.77, *SE* = 0.27, in trials with correct‐fast responses (after square: 4.74, after angry: 4.75, after happy: 4.81) and *M* = 4.80, *SE* = 0.27, in trials with correct‐slow responses (after square: 4.66, after angry: 4.92, after happy: 4.83). The average rating given to happy faces was *M* = 4.54, *SE* = 0.26, in trials with correct‐fast responses (after square: 4.64, after angry: 4.53, after happy: 4.46) and *M* = 4.48, *SE* = 0.26, in trials with correct‐slow responses (after square: 4.45, after angry: 4.42, after happy: 4.58). The ANOVA did not show any significant effect *F*s < 2.20, *p*s > 121. The absence of any significant effect in the participants’ judgments indicated that performance did not influence the perceived emotional strength of both angry and happy faces.

## 4 DISCUSSION

This experiment investigated whether the detection of an optimal response based on internal signals evokes physiological arousal and whether the identification of such an ideal performance affects the processing and evaluation of subsequent emotional faces. Participants performed a response‐choice task and received either informative (colored squares) or uninformative (happy or angry faces) feedback of performance quality. The primary outcome of the present experiment was an augmented SCR in trials with correct‐fast responses. This outcome indicates that the detection of an optimal performance based on internal signals evokes physiological arousal. At the brain electrophysiological level, in agreement with Valt and Stürmer (2017), the results showed that correct responses elicited a more negative CRN when they were fast compared to when they were slow. The detection of a correct‐fast performance did not affect only the monitoring of internal signals but influenced the processing of emotional faces, presented as uninformative feedback of performance, as well. The EPN evoked by emotional faces was less negative in trials with correct‐fast responses compare to trials with correct‐slow responses (see also, Valt & Stürmer, 2018), and this effect applied to both happy and angry faces. However, despite the significant electrophysiological effect of performance on face processing, optimal and suboptimal responses did not induce significant modulations of the subjective evaluation of the emotion. To summarize, the electrophysiological and electrodermal results corroborate the hypothesis that the detection of an optimal response based on internal signals generates arousal and affects the brain processing of emotional material, independently of its valence.

Previous studies on error monitoring found an association between electrophysiological and electrodermal activity. Incorrect responses elicit an enhanced response‐locked negative potential over medial fronto‐central recording position and, at the same time, an increase of arousal, measured as phasic SCR (Hajcak et al., 2003; Paul et al., 2017). The present experiment replicated the observation of increased response‐related negativity and arousal after errors and extended this result by showing that, compared to suboptimal responses (correct‐slow), optimal responses (correct‐fast) were linked to stronger response‐related negativity and arousal as well. The present SCR results seem to describe an association between the monitoring of correct responses based on internal signals and arousal because the observed SCR modulations neither reflected arousal evoked before the response nor arousal elicited by informative feedback after the response. Studies have shown that the SCR is a slow activity that requires a minimum of 1 s to build‐up (Benedek & Kaernbach, 2010). The observation of significant SCR modulations related to response speed mainly in the late time window (2.0–3.5 s) conforms to the timing of the effects observed by Hajcak et al. (2003) and is in line with the interpretation that the onset of arousal occurred after, and not before, the response. Hence, SCR outcomes support the hypothesis that this result reflected a phasic reaction to the response and not the effect of an ongoing tonic state of arousal. Moreover, in the SCR analysis, effects from the preceding trial were significant in the early time window but not in the late time window underlining the separation between SCR modulations linked to effects before the response, like the feedback in the previous trial, and SCR modulations evoked by the responses in the current trial. However, despite the missing interaction between performance and preceding feedback, the request for a manual response after faces but not after colored squares imposed an experimental confound that does not allow ruling out the contamination of the results by residual motor factors.

In the present experiment, we checked for the possibility that the observed performance‐related SCR modulation was driven by external signals. Colored squares were informative feedback of performance, whereas happy and angry faces were unrelated to performance. Therefore, in trials with colored squares, internal and external signals were both informative sources for performance monitoring, whereas in trials with emotional faces, internal signals were the only source for performance monitoring. The presence of a significant SCR effect when the analysis considered only trials with uninformative feedback indicated that the triggering of arousal does not need an informative external signal, as long as the monitoring system can detect an optimal performance based on internal signals. To summarize, the present SCR results are coherent with the interpretation that the detection of an optimal response based on internal signals generates arousal.

Reverting to the brain electrophysiological response, the response‐related negativity evoked over medial fronto‐central recording positions was larger for incorrect (Ne/ERN) than correct (CRN) responses; but among correct responses, optimal responses evoked a more negative potential than suboptimal response (see Valt & Stürmer, 2017). Hence, internal signals for performance monitoring are sensitive to both response accuracy and speed, inducing an augmented brain activity for both incorrect and optimal responses. The similarity between the response‐locked electrophysiological activity and the electrodermal activity seems to indicate that both responses might reflect the same process that is independent of the positive or negative valence of correct‐fast and incorrect responses, respectively. However, the absence of a correlation between ΔSCR and ΔCRN shows that the two processes might co‐occur without being tightly related. Compared to correct‐fast responses, errors evoked a significantly larger electrodermal and electrophysiological response, suggesting that the reaction of the monitoring system to errors is stronger compare to the reaction induced by optimal responses. Importantly, errors were significantly slower than correct‐fast responses, meaning that the pattern of response‐related amplitudes did not describe the possibility that errors and correct‐fast responses were just the effects of a chance response that was correct in some trials and incorrect in other trials. Moreover, the exclusion of responses with RT faster than 250 ms further limited this potential confound from chance responses.

Besides the link between monitoring of internal signals and arousal, we observed an effect of performance also in the processing of faces presented as uninformative feedback of response speed. The EPN evoked by angry and happy faces was diminished for faces that appeared after a correct‐fast response. This result replicates and extends the previous observation that correct‐fast responses induce a modulation of the EPN evoked by smiling faces (Valt & Stürmer, 2018). Since in the present experiment faces appeared directly after the response, the significant effect of performance on the amplitude of the EPN indicates that the identification of a correct‐fast response based on internal signals is sufficient to induce modulations on the processing of an unrelated facial stimulus, without the need of further support from external feedback. Moreover, the observation of similar effects of performance on happy and angry faces suggests that, contrary to the negative connotation of errors, the effect induced by optimal responses is independent of valence. The observation of modulations for both angry and happy faces conforms to the concept that arousal is a valence‐unspecific state of physiological activation (Russell, 1980). However, since the EPN describes a negativity elicited by emotional faces, when contrasted against neutral faces, the observation that the physiological activation induced by correct‐fast responses did not enhance but reduced this negativity makes the interpretation of this result according to emotion modulation difficult. Moreover, the absence of any significant difference in the emotionality judgment is an additional clue that the recorded performance‐related modulation in face processing might not reflect a change in emotion processing. Investigations on the processing of basic features of neutral faces have shown that the P2, a positive peak evoked over parieto‐occipital electrodes at around 200 ms after face onset, is sensitive to changes in face configuration (Itz et al., 2014; Mercure et al., 2008). Therefore, the present effect might reflect an enhanced P2 instead of a reduced EPN, indicating a boost in the processing of second‐order features, like configuration information. Future research is nevertheless required to understand the functional meaning of the observed performance‐related modulation of face processing.

The present experiment aimed to test physiological arousal in an experimental design that used different feedback conditions. Although the performed statistics suggest that the significant effects of performance did not depend on feedback processing in the current trial or residual effects from the previous trials, a task without feedback and longer delays between trials would have allowed a more detailed EDA analysis and a better characterization of its temporal dynamics. This aspect is a substantial limitation in the present experiment. An additional limitation of the present study is the association of a small monetary punishment to correct‐slow responses. This procedure might have invited participants to treat correct‐slow responses as errors. However, the results seem to contradict such a hypothesis because, otherwise, we should have observed larger activation for correct‐slow responses than correct‐fast responses. Moreover, although in Valt and Stürmer (2017) participants preferentially judged their response speed as average, in this experiment, we did not collect subjective judgments of expected performance quality and we, therefore, cannot be sure that correct‐fast responses are actually better‐than‐expected for all the participants.

## 5 CONCLUSION

The present study shows (a) that internal signals are sensitive to processing response speed, (b) that the detection of a correct‐fast response induces phasic arousal and (c) affects the processing of emotional faces, irrespective of the positive or negative valence of the expressed emotion. These effects could reflect a predisposition of the monitoring system to detect and reinforce correct and especially fast responses to support the learning of optimal performances. According to the reinforcement‐learning model of Holroyd and Coles (2002), the Ne/ERN reflects the transmission of a dopaminergic signals from mesencephalic brain regions to the anterior cingulate cortex (ACC) as a negative reinforcement calling for the modification of responses that are worse than expected, like errors. Within this framework, the observation of a more negative CRN for correct‐fast responses could reflect a dopaminergic response elicited by optimal responses as a positive reinforcement of performances that are better than expected, leading to a state of physiological activation. This interpretation finds theoretical support in the seminal work of Schultz et al. (1997) on the activity of dopamine neurons in monkeys. They observed an increase in the activity of dopamine neurons when an event was better than expected and a decrease when an event was worse than expected. Hence, the dopaminergic activity ​might describe violations of predictions, both when they are positive, like an optimal response, or negative, like an error, and the ACC might react to such changes of dopaminergic activity. Within this framework, the response‐related negativity might reflect the detection of a change in the dopaminergic signal that results in enhanced CRN for better‐than‐expected responses (correct‐fast) and the Ne/ERN for worse‐than‐expected responses (errors). This interpretation finds additional support from the observation that both positive and negative unexpected feedback evokes enhancements of the feedback‐related negativity (Ferdinand et al., 2012; Oliveira et al., 2007). The FRN is considered the feedback‐locked counterpart of the Ne/ERN (Miltner et al., 1997). An enhanced FRN in response to unexpected positive and negative feedback suggests that the monitoring system reacts to deviations from expected outcomes, and the enhanced CRN might reflect a deviation from the average performance according to response speed, as the Ne/ERN might signal a deviation according to accuracy. To conclude, the monitoring system is sensitive to both optimal performances and errors. Such sensitivity might be an essential feature for directing learning toward perfection.

## CONFLICT OF INTEREST

The authors declare no conflict of interest.

### PEER REVIEW

The peer review history for this article is available at https://publons.com/publon/10.1002/brb3.2162.

## Data Availability

The data that support the findings of this study are available from the corresponding author upon reasonable request.
